# Deep Neural Networks for Automatic Atrial Fibrillation Detection Using Long-Term Ambulatory Electrocardiography: Retrospective Diagnostic Accuracy Study

**DOI:** 10.2196/83714

**Published:** 2026-06-30

**Authors:** Jagdeep Sedha, Jukka A Lipponen, Antti Aho, Helena Jäntti, Onni E Santala, Tomi P Laitinen, Tiina M Laitinen, Jari Halonen, Mika P Tarvainen, Eemu-Samuli Seljola, Noora S Naukkarinen, Olli Rantula, Tuomas T Rissanen, Juha E K Hartikainen, Tero J Martikainen

**Affiliations:** 1 School of Medicine, Faculty of Health Sciences, University of Eastern Finland, Yliopistonranta 8, Kuopio, 70210, Finland, 358 443358523; 2 Heart Center, Kuopio University Hospital, Kuopio, Finland; 3 Department of Technical Physics, University of Eastern Finland, Kuopio, Finland; 4 Department of Anesthesiology and Intensive Care, North Karelia Central Hospital, Joensuu, Finland; 5 Department of Clinical Physiology and Nuclear Medicine, Kuopio University Hospital, Kuopio, Finland; 6 Mikkeli Central Hospital, Mikkeli, Finland; 7 University of Helsinki, Helsinki, Finland; 8 Heart Center, North Karelia Central Hospital, Joensuu, Finland; 9 Department of Prehospital Emergency Care, Acute Services, Kuopio, Finland

**Keywords:** atrial fibrillation, deep neural networks, electrocardiogram, electrocardiogram analysis, ECG analysis, cardiac arrhythmia detection, artificial intelligence, AI, machine learning

## Abstract

**Background:**

Atrial fibrillation (AF), the most prevalent cardiac arrhythmia, affects 2% to 4% of the global adult population and is associated with an increased risk of stroke. Early diagnosis of AF and atrial flutter (AFL) is crucial due to their association with stroke risk and the challenge posed by their often asymptomatic and episodic nature. Traditional electrocardiogram (ECG) interpretation requires substantial expert input and can be challenging, especially with poor-quality ECGs.

**Objective:**

This study aimed to evaluate the performance of a deep neural network (DNN) model in detecting AF/AFL from a large, heterogeneous set of long-term ambulatory ECG recordings, including clinical data collected over 6 months at a university hospital, and assess its effectiveness in a setting reflecting the diversity and complexity of real-world clinical data.

**Methods:**

The research combined public datasets totaling 10,248 patients, ECG data from our previous studies (648 patients), and authentic long-term ECG recordings from 4346 patients at Kuopio University Hospital for development of the DNN model. Its clinical accuracy and generalizability were assessed using a separate test dataset consisting of 1039 pseudonymized long-term ECG recordings from 1010 patients, all thoroughly reviewed and annotated by experts.

**Results:**

The DNN model demonstrated high effectiveness, achieving 96.4% sensitivity and more than 99.99% specificity for time-level AF and AFL detection. At the recording level, it identified AF and AFL with 100% sensitivity and 98.77% specificity, producing false positives in only 1.2% (11/897) of recordings, of which 81.8% (9/11) had other non–AF/AFL arrhythmias. The model maintained high performance across diverse patient characteristics, including varying ages, comorbidities, coexisting arrhythmias, and poor-quality ECG recordings.

**Conclusions:**

The results demonstrate that the proposed DNN model may support automated screening for AF and AFL in long-term ambulatory ECG recordings and may reduce manual review workload in clinical practice.

## Introduction

Atrial fibrillation (AF) is the most prevalent cardiac arrhythmia, affecting 2% to 4% of the global adult population [1]. AF, as well as atrial flutter (AFL), is associated with an increased risk of stroke and substantial health care costs [1]. The prevalence of AF and AFL is predicted to increase 2.3-fold by 2060 due to the aging population [1-4]. The diagnosis of AF and AFL requires electrocardiogram (ECG) documentation. However, AF and AFL are often intermittent and asymptomatic. Thus, standard 24- or 48-hour ECG recordings are often insufficient for detecting AF and AFL, making early detection and prevention challenging [5-7].

AF is characterized by disorganized atrial electrical activity, resulting in an irregular ventricular response and absence of discrete P waves on surface ECG recordings [8-11]. In contrast, AFL is a more organized atrial arrhythmia, typically driven by a macroreentrant circuit and associated with regular flutter waves [12]. Although AF and AFL share common risk factors and may coexist, their ECG manifestations differ in ways that are relevant for automated rhythm detection [9-11].

From an automated ECG analysis perspective, AFL presents a particular challenge due to variable atrioventricular (AV) conduction. Irregular ventricular responses in AFL can closely resemble those of AF on surface ECG, leading to frequent misclassification by both rule-based and deep learning–based methods [13-15]. As a result, approaches relying primarily on RR interval irregularity may struggle to distinguish AFL with variable AV conduction from AF [6-8].

AF may be classified as paroxysmal or persistent based on episode duration and termination characteristics [16,17]. Paroxysmal AF is intermittently present and, therefore, more likely to be missed in long-term ambulatory ECG recordings, whereas persistent AF is continuously detectable [16,17].

In addition to true atrial arrhythmias, several cardiac rhythm disturbances such as supraventricular ectopic beats (SVEBs), AV conduction abnormalities, and atrial tachycardias may mimic AF or AFL on surface ECG, increasing the risk of false-positive detections in automated ECG analysis [18,19].

Recently, longer ECG recordings lasting a week or even longer have become available owing to the emergence of novel ECG recording devices such as wearable single-channel ECG devices [20,21]. However, longer ECG recordings typically also encompass 10% to 30% of noisy data and artifacts [22,23] arising from motion-related interference or muscular activity [20,21]. Consequently, contemporary analysis techniques require substantial manual effort from health care personnel, particularly when analyzing noisy ECG data [20,21]. Given the growing incidence of cardiac arrhythmias and the increasing duration of recordings, there is an unmet demand for automated approaches capable of accurate detection of cardiac arrhythmias [20,21].

In contrast to the traditional stepwise method of automated arrhythmia analysis involving signal preprocessing, feature extraction, and classification [24], our study focused on the application of deep neural networks (DNNs). These networks offer comprehensive, end-to-end processing of raw ECG data [21], potentially uncovering new diagnostic features from large volumes of unprocessed data.

Several end-to-end DNN models have been proposed for ECG-based AF detection [25-34] and arrhythmia analysis [7,21]. Most of these models have been developed and evaluated using small public databases with a limited number of recordings. This limitation hampers the development of models that are generalizable, resulting in poor performance when applied to unseen data. While a few recent studies have applied DNNs to long-term ambulatory recordings, they have not assessed performance at the full recording or patient level in real-world clinical datasets [35,36].

Furthermore, only a few models have been designed and tested using a substantial amount of ambulatory data, which are often affected by various artifacts, other rhythm disorders, and noise that can mimic AF [25,37,38]. The application of these DNN models for AF screening in ambulatory, free-living conditions remains an open challenge [11]. Even the most advanced DNN methods developed with substantial amounts of ECG data [28-30,35,36] still face a significant issue: a high rate of false positives when applied to patient-operated ambulatory ECGs collected under real-world conditions.

In this study, we used an extensive set of authentic clinical data and specifically investigated the capability of a DNN model for automatic identification of patients with AF and AFL based on 24- and 48-hour ECG recordings while highlighting pertinent ECG segments exceeding 30 seconds for physician review.

## Methods

### Training and Validation Data

**Figure 1.**
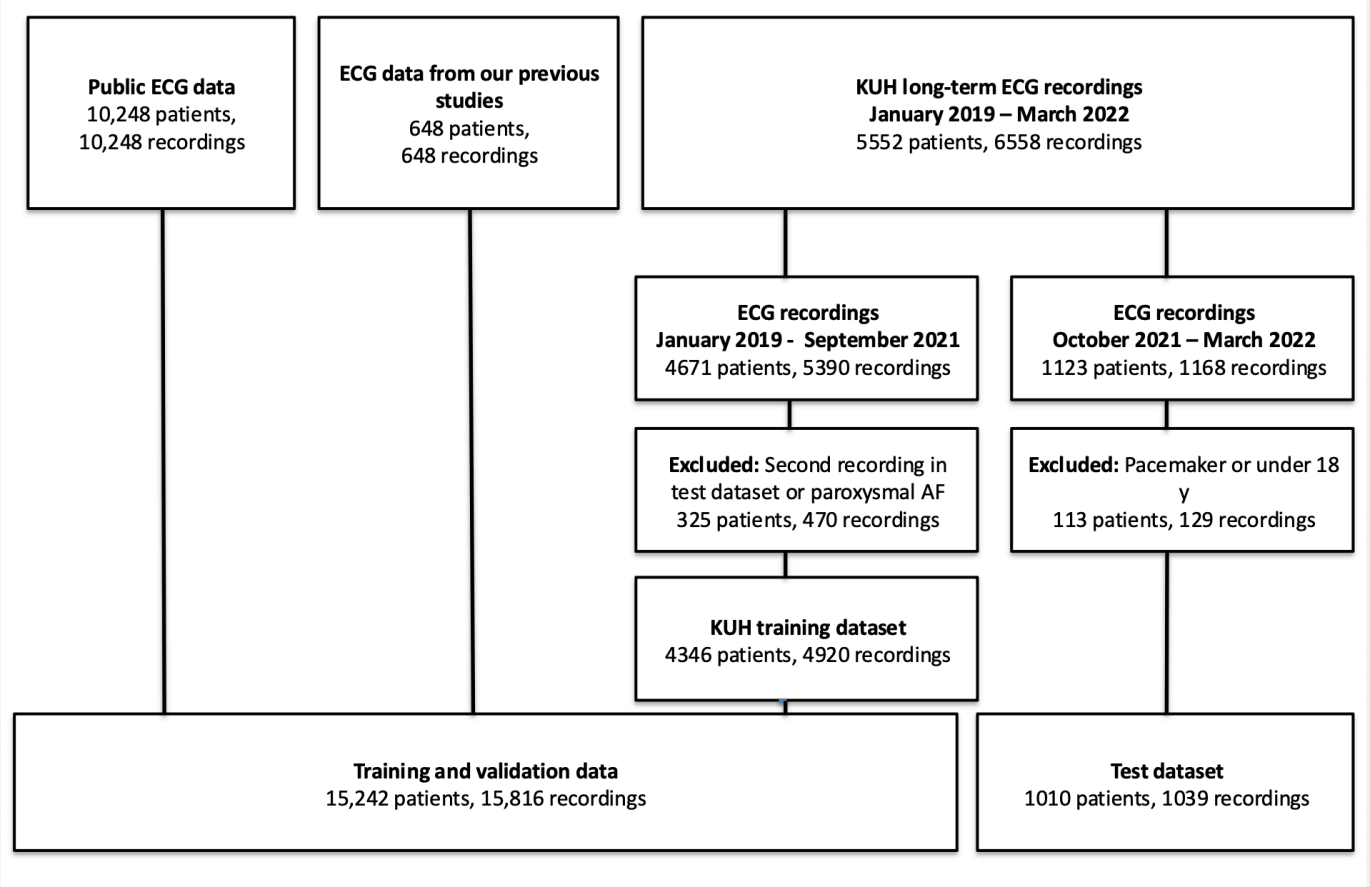
Study flowchart for electrocardiogram (ECG) recordings illustrating the division into training and validation data and a separate test dataset, with exclusions noted. Patient counts in the period-specific KUH subsets are not mutually exclusive because some patients had ECG recordings in both time periods. These patients are counted in both period-specific boxes but only once in the total KUH patient count. AF: atrial fibrillation; KUH: Kuopio University Hospital.

The training and validation dataset comprised 10,896 recordings from 10,896 patients. Of these, 10,248 recordings were gathered from a public ECG dataset, while the remaining 648 recordings were obtained from our previous studies designed for training and validation of machine learning methods for arrhythmia detection (Figure 1, Table 1). The flowchart illustrating the training and validation data is presented in the Results section. Public datasets provided AF and AFL labels that were used as is, whereas ECG recordings from previous studies were annotated by trained physicians. Additionally, the training and validation dataset included samples from long-term ECG recordings at Kuopio University Hospital (KUH). The KUH training dataset encompasses recordings from January 2019 to September 2021 (4920 recordings from 4346 patients). Clinical ECG data from KUH were labeled according to standard clinical practice and analyzed using the Medilog Darwin software (Schiller). During the clinical review process, arrhythmic events, including AF and AFL, were identified and verified by experienced ECG analysis specialists. In addition, ectopic beats were classified as ventricular ectopic beats or SVEBs and verified during expert review. ECG segments in which all 3 channels were too noisy for reliable identification of QRS complexes were labeled as nonanalyzable. Recordings from patients with paroxysmal AF were excluded from the training and validation data and moved to the KUH test dataset (see below). In addition, patients who were identified in the KUH test dataset were excluded from the KUH training and validation dataset. Across all training datasets, including public datasets, the total number of patients was 15,242, of whom 3180 (20.9%) had AF or AFL.

**Table 1.** Overview of training and validation datasets detailing the number of electrocardiogram (ECG) recordings, the number of patients per dataset, and the duration of ECG recordings for each dataset.

Database	Number of recordings (n=15,816), n (%)	Number of patients (n=15,242), n (%)	Recording duration
KUH^a^ training dataset	4920 (31.1)	4346 (28.5)	24 h
ECG data from our previous studies			
Arrhythmia detection and 1-time ECG [39,40]	461 (2.9)	461 (3.0)	0.5-1 h
AFIB24h^b^ dataset [41]	187 (1.2)	187 (1.2)	24 h
Public ECG data			
Long-Term AF^c^ Database [42]	84 (0.5)	84 (0.6)	24 h
Chapman University dataset [43]	10,000 (63.2)	10,000 (65.6)	10 s
Smart Health for Assessing the Risk of Events via ECG dataset Database [44]	139 (0.9)	139 (0.9)	24 h
MIT^d^-BIH^e^ Atrial Fibrillation Database [45]	25 (0.2)	25 (0.2)	10 h

a KUH: Kuopio University Hospital.

b AFIB24h: Atrial Fibrillation Detection: 24 Hour Study.

c AF: atrial fibrillation.

d MIT: Massachusetts Institute of Technology.

e BIH: Beth Israel Hospital.

Altogether, the training and validation data contained ECG samples from 15,242 patients obtained using various ECG recorders and from different geographical areas. A randomly selected 20% of the training dataset recordings were used as a validation dataset to perform DNN hyperparameter tuning. The training, validation, and test datasets had separate sets of patients. The initial split was performed at the recording level. Subsequently, all recordings from patients appearing in the test set were removed from the validation or training sets, and recordings from patients appearing in the validation set were removed from the training set. As a result, the final training, validation, and test datasets had mutually exclusive patient groups, whereas the test dataset could contain multiple recordings from the same patient. Public datasets were derived from independently collected cohorts in the United States, China, and Italy, whereas the local dataset was collected at KUH. Given the independent origins of these datasets, patient overlap between sources was not expected, thereby reducing the risk of data leakage across training, validation, and test sets.

### Test Dataset

Clinical accuracy and the generalizability of the method were tested using 1039 retrospective pseudonymized long-term ECG recordings from 1010 patients (test dataset) conducted at KUH between October 2021 and March 2022. The exclusion criteria were pacemaker use (n=24, 2.3% of the recordings) and patients under 18 years (n=105, 10.1% of the recordings). Arrhythmias underwent electrophysiologist review including arrhythmia classification identified during clinical practice analyzed using the Medilog Darwin software. In addition, segments whose 3 channels were too noisy for identification of QRS complexes were assessed as nonanalyzable. ECG recordings in the test datasets underwent an additional rhythm annotation procedure where researchers verified and corrected (if needed) on a second-by-second basis all AF and AFL segments. Rhythm annotations made during clinical practice were used as a starting point. The AF class combined both AF and AFL rhythms. Medication data were obtained through automated extraction from the hospital electronic health record system at KUH.

### Development of the DNN Model

We developed a convolutional DNN model specifically designed for the detection of AF and AFL. We selected a residual network as the core architecture, incorporating shortcut connections between every second convolutional layer to address the vanishing gradient problem [46,47]. A similar residual network has been successfully applied to an ECG arrhythmia classification task before [21].

The model processes a 10-second segment of raw ECG data as input, consisting of 3 channels sampled at 125 Hz, and requires no additional patient-related information. The model classifies the 10-second ECG input into 3 categories: AF or AFL, non–AF/AFL, or nonanalyzable. Segment-level classification was performed using the maximum of the output without class-specific probability thresholds. The number of convolutional filters was doubled after every 4 convolutional layers [21]. Prior to each convolutional layer, batch normalization and a rectified linear unit activation were applied [48].

The network weights were initialized using the initialization method by He et al [49]. For optimization, the Adam optimizer [50] was used with a batch size of 128 and an initial learning rate of 10^–3^. Additionally, dropout with a probability of 0.1 was used to prevent overfitting [51]. The model was trained using a categorical cross-entropy loss. To improve training efficiency, the learning rate was reduced by a factor of 10 if the validation loss did not improve for 2 consecutive epochs.

Model hyperparameters—including the number of convolutional layers (search space: 17 to 37), number of filters (8 to 32), filter size, and dropout rate (0 to 0.4)—were optimized using grid search. The model with the lowest validation loss during training was selected for final evaluation.

The final optimized network consists of 37 convolutional layers. The convolutional layers initially use 16 filters with a filter length of 12, and the number of filters increases after every 4 layers. A schematic overview of the DNN architecture is presented in Figure S1 in Multimedia Appendix 1.

The model output was subsequently postprocessed to account for the temporal continuity of detected rhythms. AF detections shorter than 30 seconds were excluded as clinically insignificant in accordance with current European Society of Cardiology guidelines defining clinically relevant AF episodes as lasting at least 30 seconds [2]. Closely spaced, clinically significant AF and AFL segments were merged into unified episodes using a 4-minute window to enhance detection sensitivity and provide more meaningful information for clinical interpretation.

### DNN Model Evaluation

All 1039 long-term ambulatory recordings in the test dataset were segmented into fixed, nonoverlapping 10-second windows and input into the DNN model. Each segment was scaled to a fixed range between −1 and 1 to reduce amplitude variability across recordings. The DNN produced 1 rhythm classification per 10-second segment (AF or AFL, non–AF/AFL, or nonanalyzable) based on signal quality and rhythm characteristics, with no segments excluded from analysis. As ECG recordings are annotated to true classes second by second, model accuracy can be assessed at the time and recording level. Time-level accuracy of AF and AFL detection was evaluated by comparing annotations to the closest prediction and deemed to be correctly classified if the annotation and prediction matched. At the recording level, patients with AF or AFL were deemed correctly detected (true positive) if there was at least one correctly detected 30-second AF or AFL segment. Patients without AF or AFL were correctly identified (true negative) if the DNN model did not predict AF or AFL in any 30-second sequence for the patient. Evaluation at the recording level is useful for assessing how the DNN model can be applied to the analysis of online ECG recordings and notify health care personnel of whether AF or AFL is detected from the ongoing recording. The time-level evaluation is similar in clinical applications such as offline ambulatory ECG analysis, where it is critical to identify the onset and offset of an AF or AFL segment.

### Ethical Considerations

This study was approved by the ethics committee of the Northern Savo Hospital District (2061/2020). This study was conducted according to the principles of the Declaration of Helsinki. No informed consent from patients was needed due to the retrospective nature of the study. All data were pseudonymized before analyses. This study used retrospective ECG data owned by KUH and was conducted as part of institutional research activities. The manuscript and supplementary materials do not include images or other content that would allow for the identification of individual participants.

## Results

### Overview

**Figure 2.**
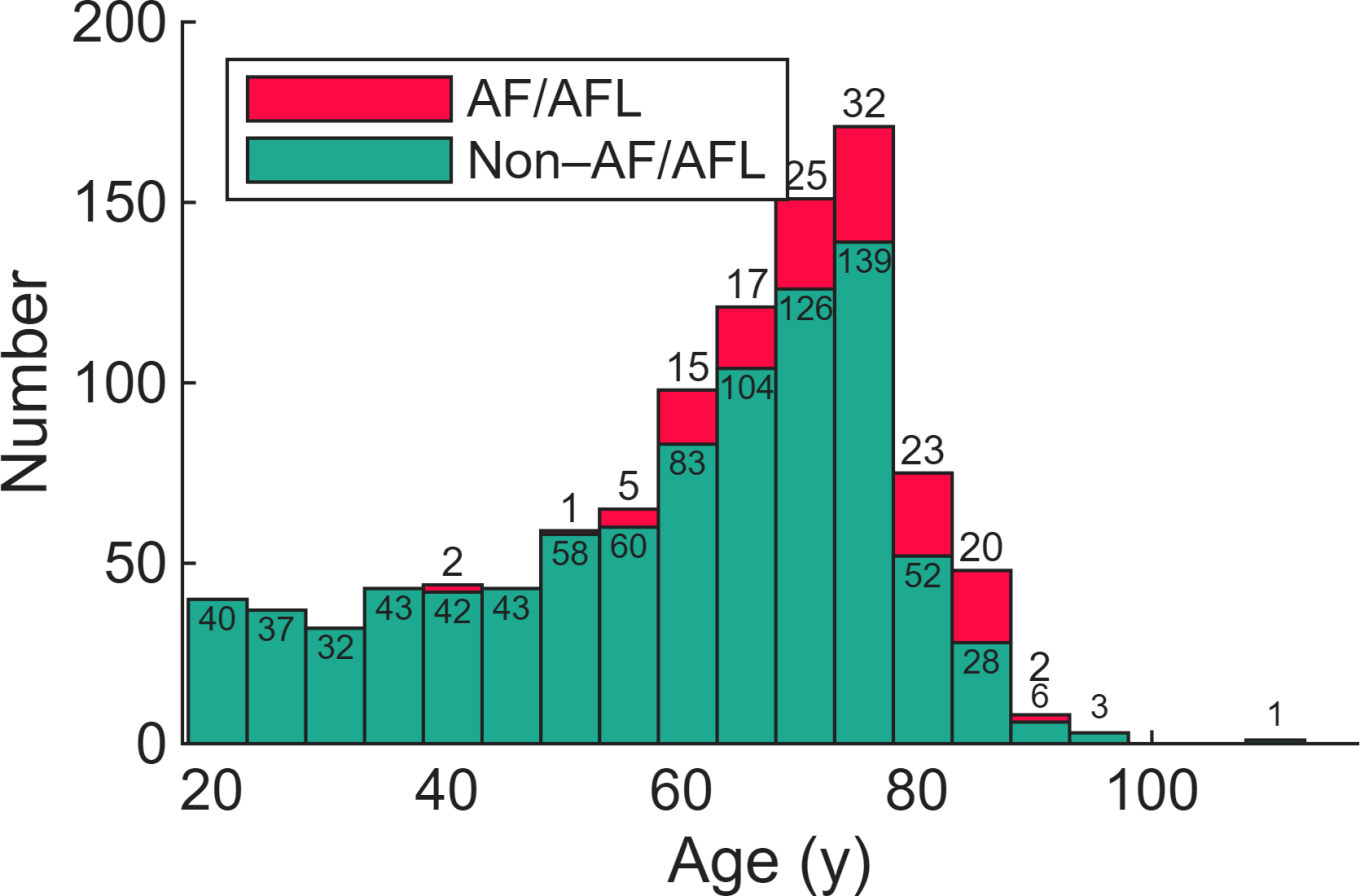
Age distribution of the test set (n=1039) recordings. The red bars denote patients with atrial fibrillation (AF) or atrial flutter (AFL), and the green bars denote patients with non–AF/AFL.

The final test dataset consisted of 1039 three-channel ECG recordings ranging from 24 to 48 hours in duration from 1010 unique individuals, as shown in the Figure 1 flowchart. The mean age of the patients was 60.37 (SD 17.75; range 18-105) years, and 48% (485/1010) were men. AF or AFL was present in 13.7% (142/1039) of the ECG recordings, of which 16.2% (23/142) exhibited both AF and AFL and non–AF/AFL arrhythmias. Figure 2 shows the age distribution and rhythm distribution of the test dataset. In total, there were 25,072 hours of ECG data, with 3028.8 (12.1%) hours of AF and AFL. AF and AFL were most prevalent in individuals aged 60 to 90 years. In addition, 0.4% (92.9/25,072) of hours of data were deemed nonanalyzable.

Data on medication were available for 66.6% (673/1010) of the dataset patients (Table 2). Notably, oral anticoagulants were used in 51.5% (50/97) of patients in the AF and AFL group. Antihypertensive drugs were the most used medication in both the AF and AFL group and non–AF/AFL group. Non–AF/AFL rhythms are shown in Table 3. Cardiologist statements on arrhythmias were available for 88.1% (915/1039) of the recordings. Notably, combined cases of ventricular ectopic beats (>1000) and SVEBs (>1000) accounted for 28.2% (258/951) of the non–AF/AFL arrhythmias. Other significant observations included supraventricular tachycardias, which were present in 17.8% (163/915) of patients, and sinus bradycardia, which was present in 7.3% (67/915) of patients.

**Table 2.** Medication use in test dataset recordings.

	AF^a^ or AFL^b^ (n=97 recordings), n (%)	Non–AF/AFL (n=576 recordings), n (%)
Data available	97 (68.3)	576 (64.2)
No medication	0 (0)	38 (6.6)
Antiarrhythmic drugs	4 (4.1)	9 (1.6)
Antihypertensive drugs	73 (75.3)	417 (72.4)
Anticoagulants	50 (51.5)	59 (10.2)
Antithrombotic drugs	8 (8.2)	101 (17.5)
Dyslipidemia	34 (35.1)	205 (35.6)
Diabetes oral drugs	11 (11.3)	56 (9.7)
Insulin	0 (0)	19 (3.3)

a AF: atrial fibrillation.

b AFL: atrial flutter.

**Table 3.** Prevalence of non–atrial fibrillation or atrial flutter arrhythmias in the test dataset categorized by type and count, with corresponding percentages of the total cases. The table includes data on ventricular and supraventricular ectopic beats (SVEBs), atrioventricular blocks (AVBs), sinus bradycardia, and other arrhythmias identified in the patient population.

Finding	Recordings (N=1039), n (%)
Data available	915 (88.1)
VEB^a^—>100	341 (37.3)
VEB—>1000	167 (18.3)
VEB—>5000	71 (7.8)
VEB—>10,000	37 (4.0)
SVEB—>100	271 (29.6)
SVEB—>1000	91 (9.9)
SVEB—>5000	23 (2.5)
SVEB—>10,000	7 (0.8)
LBBB^b^	11 (1.2)
RBBB^c^	10 (1.1)
AVB I	66 (7.2)
AVB II	33 (3.6)
AVB III	0 (0)
Junctional rhythm	8 (0.9)
SVT^d^	163 (17.8)
VT^e^	49 (5.4)
Sinus bradycardia	67 (7.3)
Sinus pause	6 (0.7)

a VEB: ventricular ectopic beat.

b LBBB: left bundle branch block.

c RBBB: right bundle branch block.

d SVT: supraventricular tachycardia.

e VT: ventricular tachycardia.

### Time-Level AF and AFL Detection

In the time-level classification (confusion matrix), the DNN model correctly classified 2918.2 hours as AF or AFL out of 3028.8 true hours of AF and AFL, resulting in 96.4% sensitivity for time-level AF and AFL detection (Table 4). The DNN misclassified 1.0 hours of non–AF/AFL data as AF or AFL and 0.1 hours of nonanalyzable ECG data as AF or AFL resulting in over 99.99% specificity, 99.96% positive predictive value (PPV), and 99.5% negative predictive value (NPV) for time-level AF and AFL detection.

**Table 4.** Comparative analysis of true vs deep neural network (DNN)–detected hours for atrial fibrillation (AF) and atrial flutter (AFL), non–AF/AFL, and nonanalyzable categories.

	DNN-detected AF and AFL (h)	DNN-detected non–AF/AFL (h)	DNN-detected nonanalyzable (h)
True AF and AFL (h)	2918.2	109.1	1.5
True non–AF/AFL (h)	1.0	21,925.0	24.3
True nonanalyzable (h)	0.1	1.5	91.3

**Figure 3.**
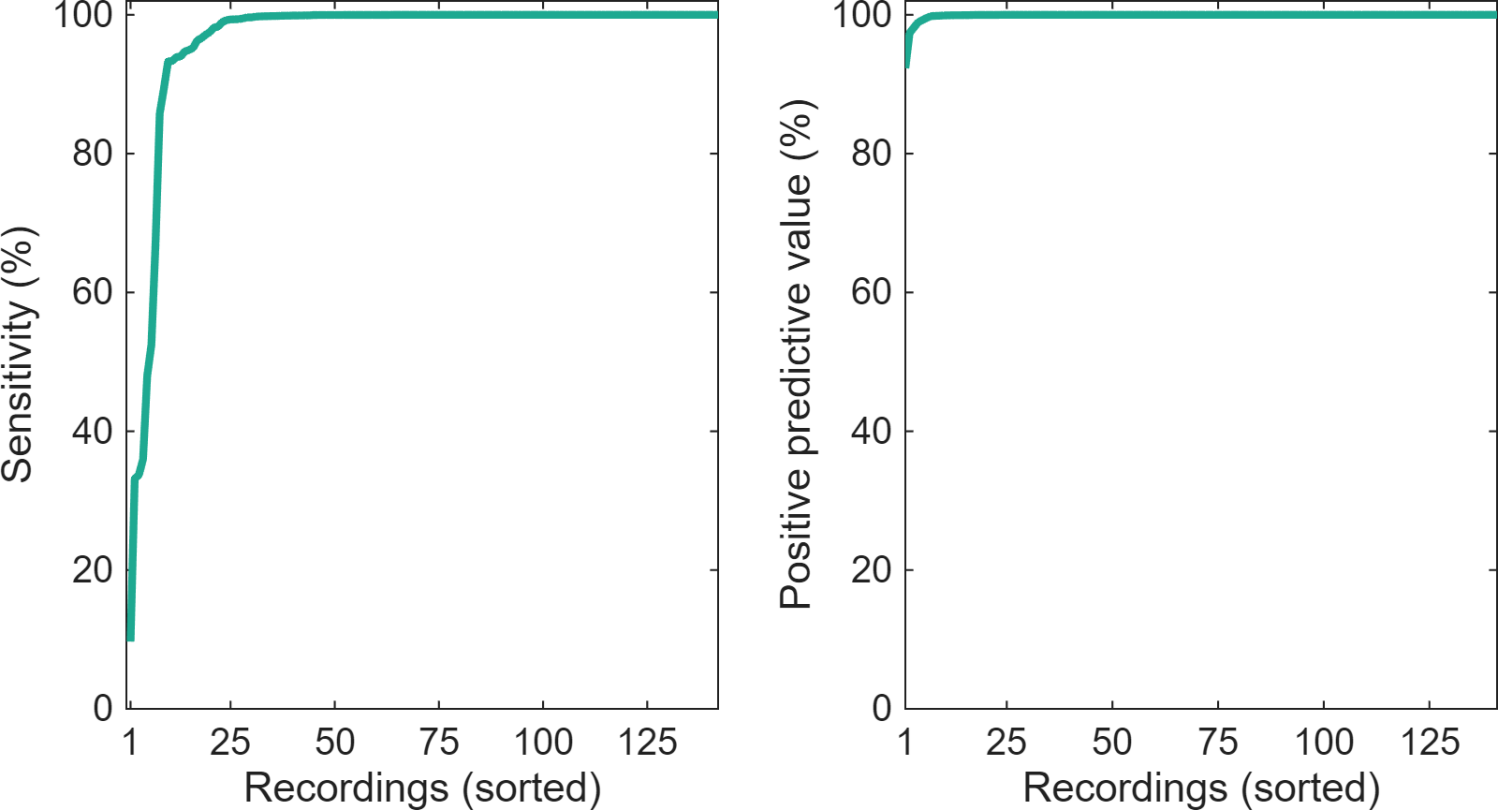
Time-level sensitivity and positive predictive value for atrial fibrillation and atrial flutter detection.

In the time-level analysis, the time-level sensitivity of the DNN model for detecting AF and AFL was greater than 90% in 93.7% (133/142) of AF and AFL recordings. For 2.8% (4/142) of the recordings, the sensitivity ranged between 50% and 90%, and for 3.5% (5/142) of the recordings, it was less than 50% (Figure 3). A detailed breakdown of the time-level analysis revealed that all 9 recordings with less than 90% time-level sensitivity had AFL rhythm with a 2:1, 3:1, or 4:1 (mainly 2:1) conduction ratio where part of the F waves were merged into the QRS complexes.

### Recording-Level AF and AFL Detection

In the recording-level detection, the DNN model detected correctly at least one AF or AFL segment of more than 30 seconds in all 142 recordings containing AF or AFL. Thus, the recording-level AF and AFL detection sensitivity was 100%.

Of the 897 recordings without AF or AFL, the DNN model generated false-positive AF or AFL predictions for 11 (1.2%), with recording-level specificity of 98.77% and PPV of 92.81%. Of these 11 recordings, 9 (81.8%) showed arrhythmias: 3 contained frequent atrial premature beats, 3 contained acute rhythm changes (eg, junctional rhythm to normal SR), and 3 contained 1- or 2-degree AV block. A total of 0.2% (2/897) of the recordings showed noisy ECGs. Recording-level sensitivity, specificity, PPV, and NPV are reported in Table 5, with 95% CIs calculated using the Wilson score method.

**Table 5.** Recording-level detection performance of the deep neural network model.

Metric	Estimate (%; 95% CI)
Sensitivity	100 (97.37‐100)
Specificity	98.77 (97.82‐99.31)
Positive predictive value	92.81 (87.59‐95.94)
Negative predictive value	100 (99.57‐100)

Nonanalyzable sections of the ECG were identified in 11.1% (115/1039) of the recordings during clinical adjudication. The total duration of nonanalyzable ECGs was 92.9 hours; most of it (58.6 hours) was derived from 0.6% (6/1039) of the recordings, where lead failure or improper skin electrode contact corrupted the ECG. The DNN model correctly classified 91.3 of the 92.9 hours of nonanalyzable ECG as nonanalyzable. In addition, the DNN model labeled 25.8 hours of data as nonanalyzable that were deemed to be analyzable in the clinical workflow. A common factor between these segments was 2 channels exhibiting large ECG artifacts, whereas only 1 channel displayed visible R peaks.

## Discussion

### Principal Findings

This study provides a comprehensive demonstration of a deep learning approach to perform automatic AF and AFL detection from a substantial dataset encompassing more than 1000 long-term ambulatory ECG recordings from real-life clinical patients. Our DNN model successfully identified all patients exhibiting AF and AFL. Notably, it generated false AF or AFL alarms in only 1.2% (11/897) of recordings of patients who did not have AF or AFL, with 81.8% (9/11) of the false alarms due to other true arrhythmias. This demonstrates that an end-to-end DNN approach has the potential to significantly enhance the precision of algorithmic interpretation for long-term ambulatory ECG analysis. We also emphasize the importance of using a sufficiently extensive test dataset in this study, allowing for the comprehensive evaluation of the DNN model’s capabilities across a diverse range of patients, including those with other arrhythmias, poor-quality ECGs, additional heart diseases, and various conditions. The training and test datasets included patients receiving a variety of rate-controlling and antiarrhythmic medications that may influence RR interval variability. The model was intentionally trained on heterogeneous real-world data without medication-specific inputs, aiming to achieve robust AF and AFL detection independent of pharmacological modulation of heart rate dynamics.

The standard approach to automated ECG interpretation involves several steps, including preprocessing the signal; extracting features; selecting or reducing these features; and, finally, classifying them [24]. In contrast, DNNs offer a fundamentally different method by allowing a single algorithm to handle all these tasks in an end-to-end manner. This means that the DNN can process raw ECG data directly and provide diagnostic probabilities without the need for specific feature extraction [21]. If trained with sufficient raw data, DNNs could potentially learn all significant features previously identified manually as well as discover new features through a data-driven approach. Indeed, our study at the recording level showed very high sensitivity and specificity for AF and AFL detection at 100% and 98.77%, respectively. The clinical relevance of these advancements is substantial given the significant amount of unprocessed clinical data used to train the DNN algorithm. The automated detection system may not only streamline the diagnostic process but could also drastically reduce manual work, enhancing efficiency in clinical workflows. Furthermore, its capability to provide real-time alerts in online measurements with an exceedingly low rate of false alarms emphasizes its potential in real-world applications.

### Comparison to Prior Work

Many prior studies examining the performance of DNN models and other methodologies for AF and AFL detection have often relied on limited ECG datasets such as the IRIDIA-AF dataset or Long-Term AF Database. These datasets typically consist of ECG recordings from a relatively small patient group [35,36,39,40]. Even DNN models developed using extensive ambulatory ECG datasets [28-30] still face a significant challenge: a high rate of false positives when applied to patient-operated ambulatory ECGs collected in real-world settings, as evidenced by a relatively low specificity of less than 99.0%. In contrast, we analyzed entire 24- to 48-hour recordings using an end-to-end DNN model that highlights clinically relevant AF and AFL segments of 30 seconds or longer for review. We evaluated its performance on a large single-center test set of 1039 recordings from real clinical patients, offering a more comprehensive and representative evaluation of DNN performance under real-world conditions. By leveraging a large and diverse training dataset collected from various ECG recorders, our model achieved comparable sensitivity while significantly improving specificity and PPV. These results highlight the potential of DNN models trained on substantial and heterogeneous datasets for improving AF detection in clinical practice.

A notable exception in long-term ambulatory ECG rhythm analysis are the studies by Zhang et al [38] and Fiorina et al [37], who evaluated DNN-based AF screening in large-scale hospital scenarios. Both DNN methods were developed using substantial ambulatory ECG datasets, yielding very promising AF screening results with a sensitivity of 99% and a specificity exceeding 98%. However, both studies focused primarily on screening patients with AF, and the capability of the DNNs to detect AF and AFL at the time level was not reported [37,38]. Importantly, our results provide strong evidence that state-of-the-art DNN models can identify patients with AF and AFL and detect AF and AFL segments in ambulatory ECGs with high temporal accuracy while effectively handling corrupted or nonanalyzable segments and maintaining a very low false positive rate regardless of the presence of other arrhythmias.

The DNN model exhibited a notably high AF and AFL time-level detection sensitivity (>90%) for 93.7% (133/142) of the patients. Upon detailed analysis, it was observed that the lower time-level sensitivity in the remaining 6.3% (9/142) of the recordings could be attributed to a specific 2:1 AFL rhythm pattern where every second P wave merged into the QRS complex. This implies that, despite its overall efficiency, the DNN model may encounter challenges in accurately identifying specific ECG patterns, as demonstrated by the 2:1 AFL rhythm. Models incorporating longer temporal context, such as convolutional neural network–bidirectional long short-term memory or transformer-based architectures, may detect transitions in AFL conduction patterns (eg, 3:1 to 2:1) and improve detection of stable 2:1 AFL segments. However, training such models would require hundreds of recordings with paroxysmal AFL and varying conduction patterns to properly capture temporal and morphological variability.

Another point of discussion is the model’s performance on recordings without AF or AFL. With a very high recording-level specificity of 98.77%, the model generated false-positive AF and AFL predictions for only 1.2% (11/897) of the recordings. In fact, 81.8% (9/11) of these false-positive cases were other true arrhythmias such as frequent ectopic beats, acute rhythm changes, and 1- or 2-degree AV block. It is also notable that only a very small percentage of these conditions caused false-positive predictions. Our model also efficiently identified nonanalyzable sections of the ECG, defined as segments in which QRS complexes could not be reliably visually verified during clinical review. No separate quantitative noise index was used. Initially, the DNN model classified only 0.1% (25.8/25072.0) of hours of data as nonanalyzable, which were marked to be analyzable in the clinical workflow. False-positive nonanalyzable labeling exceeding 10 minutes occurred in 37 of 1039 recordings. Further in-depth analysis uncovered that a significant portion of these data, totaling 10 hours, originated from seven 24-hour recordings where 2 out of 3 ECG channels manifested significant noise or lead failure. Regarding clinical consequences, AF and AFL masking due to nonanalyzable labeling was limited. As shown in the confusion matrix, approximately 0.1% (1.5/3028.8) of hours of true AF and AFL detections were classified as nonanalyzable, whereas a markedly larger proportion of true AF and AFL detections (109.1/3028.8, 3.6%) was misclassified as non–AF/AFL.

In the test dataset, the prevalence of AF and AFL was 13.7% (142/1039), which is lower than that reported in some real-world Holter monitoring populations [52] but substantially higher than that reported in opportunistic screening settings, where prevalence may be as low as 1% [53]. At this prevalence level, the model achieved a PPV of 92.81% (95% CI 87.59%‐95.94%) and an NPV of 100% (95% CI 99.57%‐100%; Table 5). As predictive values are inherently prevalence dependent, the high NPV observed in this study reflects the moderate prevalence of AF and AFL in the test set and indicates strong ability to rule out arrhythmia in this context. In lower-prevalence screening populations, PPV would be expected to decrease despite unchanged sensitivity and specificity, underscoring the importance of population-specific evaluation.

Subgroup analyses by age, sex, noise burden, and non-AF arrhythmia burden are presented in Table S1 in Multimedia Appendix 1. These analyses showed that AF and AFL detection sensitivity was lowest in recordings with a lower burden of non-AF arrhythmias, whereas both sensitivity and PPV were lowest in recordings with a high burden of non-AF arrhythmias. This was expected as other atrial and ventricular arrhythmias represent the most challenging confounders for differentiating AF and AFL in long-term ambulatory ECG recordings.

### Methodological Considerations and Limitations

Despite the promising results, our study had certain limitations. First, we excluded patients with pacemakers from the KUH dataset (Figure 1). The pacemaker inherently maintains a steady rhythm during AF and AFL. This stabilization renders the recognition of AF and AFL from a surface ECG exceedingly challenging. Pacemaker-related artifacts are a common source of false-positive AF and AFL detections in clinical practice, and their exclusion may lead to higher specificity than in a true all-comer population. Therefore, the reported performance metrics should be interpreted in the context of this limitation.

Second, the test dataset was derived from a single center (KUH), resulting in limited ethnic diversity and limited variation in ECG devices. Although the training and validation datasets included ECG recordings from multiple systems (including Schiller, Bittium Faros, GE HealthCare MUSE 12-lead ECG, and other ambulatory 3- and 4-lead recorders), the model may still be sensitive to device-specific artifacts, which could constrain external validity and generalizability across different clinical settings, recording systems, and populations; therefore, future multicenter evaluations are warranted.

Third, patients with paroxysmal AF were excluded from the KUH training and validation datasets. The model was primarily trained on persistent AF segments, which may differ to some extent from paroxysmal AF episodes. However, because the model operates on independent 10-second ECG segments without temporal context, AF and AFL detection is based on segment-level rhythm characteristics, which limits the impact of this difference.

Fourth, the model demonstrated reduced time-level sensitivity in AFL with fixed 2:1 AV conduction ratio, which represents a clinically relevant limitation. In this rhythm pattern, atrial activity may be partially obscured by the QRS complexes, making reliable discrimination from surface ECG challenging. Consequently, this specific AFL subtype constitutes a potential blind spot for automated screening.

Fifth, from a clinical safety perspective, the proposed DNN model should be regarded as a screening and decision support tool rather than a stand-alone diagnostic system. This is particularly important given the limited interpretability of the DNN models as learned features cannot be directly linked to established physiological markers; therefore, negative automated findings should be interpreted in conjunction with clinical judgment, especially when clinical suspicion of arrhythmia remains high.

Sixth, the 4-minute merge window used to ensure continuity of longer AF and AFL segments may lead to overestimation of AF burden, particularly when multiple short episodes occur close in time.

### Conclusions

The results of this study affirm the potential of using a DNN model for AF and AFL diagnostics in ambulatory settings with high sensitivity and specificity. When trained using an ample amount of data, the DNN model demonstrates the ability to distinguish ambulatory ECG changes caused by motion artifacts from AF and AFL with a low false positive rate. The presented automated detection system may streamline the diagnostic process and clinical workflows with a low false alarm rate, supporting its clinical applicability.

## Supplementary material

10.2196/83714 Multimedia Appendix 1 Deep neural network (DNN) model architecture, performance evaluation before temporal postprocessing, and analyses across demographic and signal-quality subgroups.

## Abbreviations

AF: atrial fibrillation
AFL: atrial flutter
AV: atrioventricular
DNN: deep neural network
ECG: electrocardiogram
KUH: Kuopio University Hospital
NPV: negative predictive value
PPV: positive predictive value
SVEB: supraventricular ectopic beat
