# Supplementary material for: Deep Neural Networks for Automatic Atrial Fibrillation Detection Using Long-Term Ambulatory Electrocardiography: Retrospective Diagnostic Accuracy Study
Source: JMIR Cardio. 2026 Jun 30;10:e83714. doi: 10.2196/83714 (PMC13318395; doi:10.2196/83714)
Supplement: Multimedia Appendix 1 [file cardio-v10-e83714-s001.pdf]

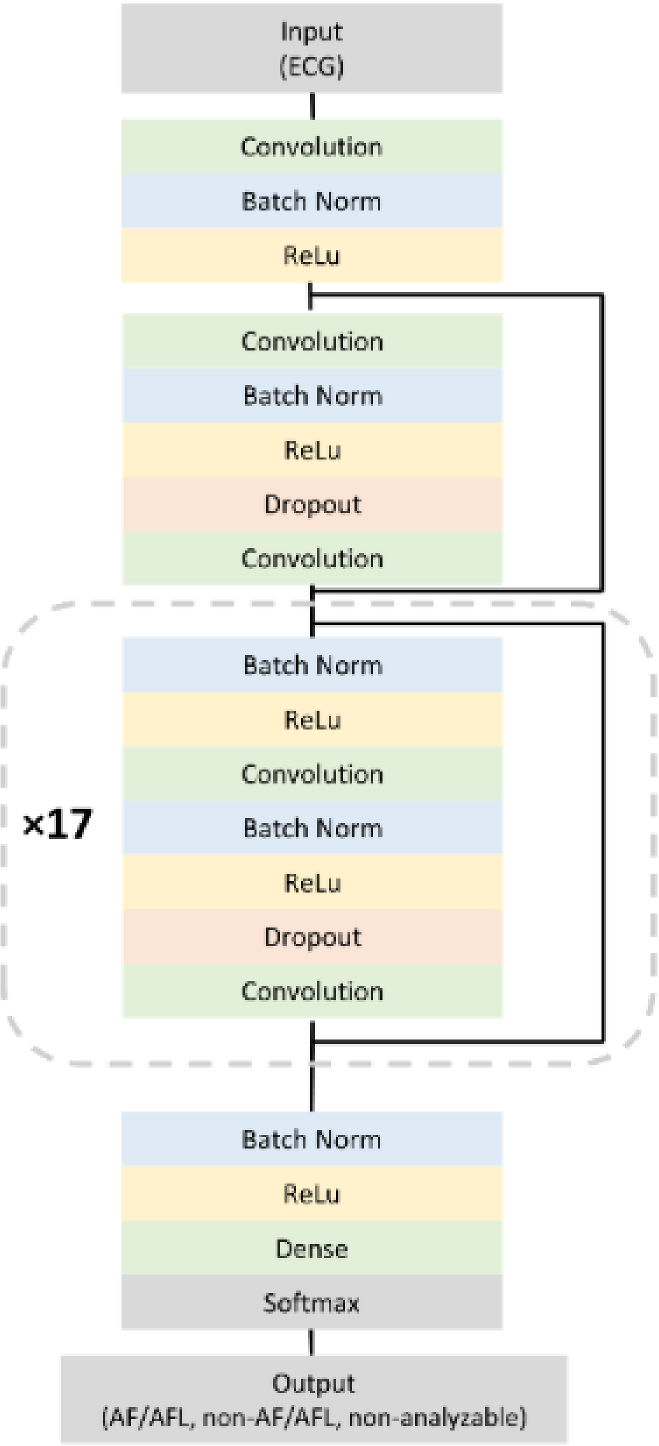

Supplementary Appendix A, Figure 1: schematic overview of the deep neural network model architecture. DNN model specifically trained for the detection of atrial fibrillation (AF) from ECG data.

Supplementary Appendix A: Model Evaluation Before Temporal Post-Processing

The time-level performance of the DNN classifier prior to temporal post-processing is summarized in Appendix Table 1 and Figure 2. Table 1 reports class-wise sensitivity, specificity, Positive Predictive Value (PPV), Negative Predictive Value (NPV), F1-score, one-vs-rest area under the receiver operating characteristic curve (AUC-ROC) and area under the precision–recall curve (AUC-PR) values for AF/AFL, non-AF/AFL, and non-analyzable segments. Figure 2 presents the corresponding confusion matrix and one-vs-rest AUC-ROC curves. The model achieved high discriminative performance across all classes, with AUC-ROC values above 0.99 and F1-scores of 96.97%, 99.54% and 86.97% for AF/AFL, non-AF/AFL and non-analyzable segments, respectively.

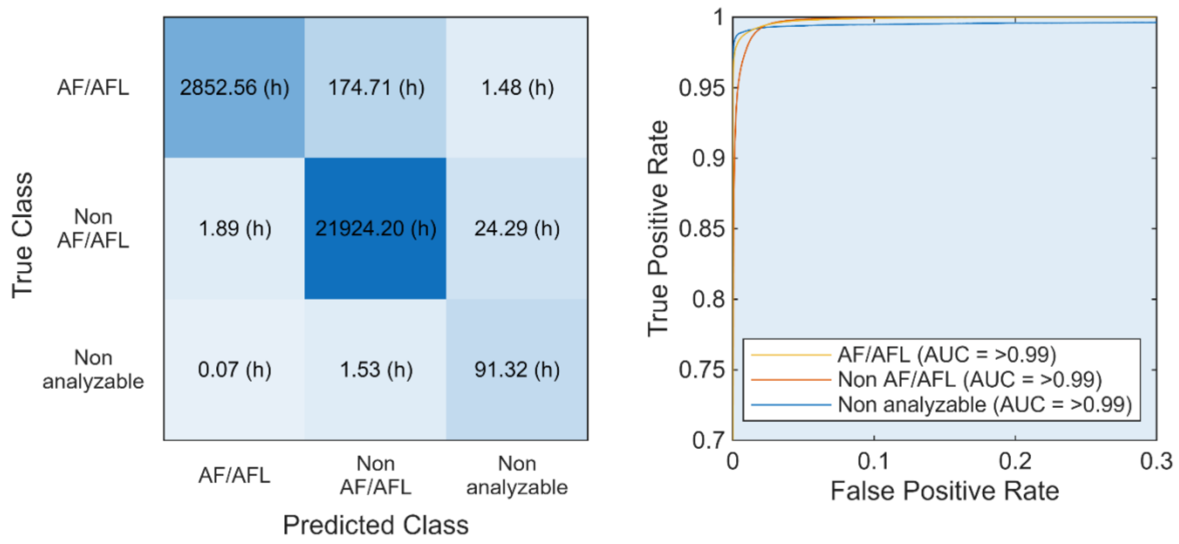

Supplementary Appendix A, Figure 2: Performance of the DNN model before incorporating temporal information. Left: Confusion matrix showing per-class classification performance expressed in hours of correctly and incorrectly classified segments for AF/AFL, non-AF/AFL, and non-analyzable rhythm categories. Right: One-vs-rest receiver operating characteristic (ROC) curves for each class

Supplementary Appendix A: Performance Across Demographic and Signal-Quality Subgroups

Supplementary Appendix A, Table 1: Time-level AF/AFL detection performance across demographic and signal-quality subgroups. The table reports the number of recordings with AF/AFL and without AF/AFL, together with sensitivity, specificity, PPV, and NPV for each subgroup: age <50 years, age ≥50 years, female, male, low arrhythmia burden, high arrhythmia burden, clean ECG, and ECG containing non-analyzable segments.

Metrics	N (AF/AFL)	N (Non AF/AFL)	Sensitivity (%)	Specificity (%)	PPV (%)	NPV (%)
Age < 50 years	2	259	100.00	>99.99	99.98	100.00
Age >= 50 years	140	638	96.29	99.99	99.95	99.30
Female	58	496	97.53	99.99	99.91	99.77
Male	84	401	95.64	>99.99	99.98	99.17
Low arrhythmia burden	93	548	95.52	>99.99	99.98	99.29
High arrhythmia burden *	49	349	98.34	99.99	99.89	99.83
Clean ECG	125	799	95.98	99.99	99.96	99.45
ECG with non-analyzable segments	17	98	99.43	99.99	99.93	99.92

* High arrhythmia burden is defined as the presence of >1000 supraventricular ectopic beats (SVEB) per day, >1000 ventricular ectopic beats (VEB) per day, or any episodes of second- or third-degree AV block, junctional rhythm, supraventricular tachycardia (SVT), ventricular tachycardia (VT), or sinus pauses.

Supplementary Appendix A, Table 2: Time level performance metrics of the DNN model before temporal post-processing.

Metrics	AF/AFL	Non AF/AFL	Non analyzable
Sensitivity (%)	94.18	99.88	98.28
Specificity (%)	99.99	94.35	99.90
PPV (%)	99.93	99.20	77.99
NPV (%)	99.21	99.12	99.99
F1-Score (%)	96.97	99.54	86.96
AUC-ROC	>0.99	>0.99	>0.99
AUC-PR	0.98	0.99	0.81

PPV = positive predictive value; NPV = negative predictive value; F1-score = harmonic mean of precision and recall; AUC-ROC = area under the one-vs-rest receiver operating characteristic curve; AUC-PR = area under the one-vs-rest precision–recall curve.
